# Effectiveness of Conservative Treatment without Early Colonoscopy in Patients with Colonic Diverticular Hemorrhage

**DOI:** 10.1155/2020/3283940

**Published:** 2020-01-14

**Authors:** Hirosato Doi, Keita Sasajima, Masanori Takahashi, Taira Sato, Iichirou Ootsu, Ryo Chinzei

**Affiliations:** Department of Digestive Internal Medicine, Saitama Red Cross Hospital, Saitama, Japan

## Abstract

**Aim:**

This study was aimed to clarify the effectiveness of conservative treatment without performing early colonoscopy and the indications for early colonoscopy in patients with colonic diverticular hemorrhage.

**Methods:**

This retrospective study included 142 participants who were urgently hospitalized due to bloody stools and were diagnosed with colonic diverticular hemorrhage between April 2012 and December 2016. At the time of hospital visit, only when both shock based on vital signs and intestinal extravasation on abdominal contrast-enhanced computed tomography were observed, early colonoscopy was performed within 24 hours after hospitalization. However, in other cases, patients were conservatively treated without undergoing early colonoscopy. In cases of initial treatment failure in patients with shock, interventional radiology (IVR) was performed without undergoing early colonoscopy.

**Results:**

Conservative treatment was performed in 137 (96.5%) patients, and spontaneous hemostasis was achieved in all patients. By contrast, urgent hemostasis was performed in five (3.5%) patients; three and two attained successful hemostasis via early colonoscopy and IVR, respectively. There were no significant differences between two groups in terms of early rebleeding (7.3% vs. 0%,*P*=0.690) and recurrent bleeding (22.7% vs. 20.0%, *P*=0.685). The factors associated with the cumulative recurrent bleeding rates were a previous history of colonic diverticular hemorrhage (hazard ratio 5.63, 95% confidence interval 2.68–12.0, *P* < 0.0001) and oral administration of thienopyridine derivative (hazard ratio 3.05, 95% confidence interval 1.23–7.53, *P*=0.016).

**Conclusions:**

In this series, conservative treatment without early colonoscopy was successful in patients with colonic diverticular hemorrhage.

## 1. Introduction

In Japan, the number of patients with colonic diverticulum has been increasing due to aging and the westernization of dietary habits, and the frequency is reportedly about one in four people [1, 2]. In addition, along with the elevated number of patients who are taking antithrombotic drugs, the prevalence of colonic diverticular hemorrhage is increasing annually, and this condition is the most frequent cause of lower gastrointestinal bleeding, accounting for approximately 25% of the total cases [3–5].

In Japanese guidelines for colonic diverticular hemorrhage, it is proposed that early colonoscopy would be preferably performed within 24 hours of hospital visit to identify the stigma of recent hemorrhage (SRH) [6], and several institutions have actively performed early colonoscopy. In addition, some new endoscopic techniques, which might contribute to increase in successful hemostasis and decrease in rebleeding, have been recently reported, such as endoscopic band ligation, endoscopic detachable snare ligation, and over-the-scope clip (OTSC®, Ovesco Endoscopy, Tübingen, Germany) [7–9]. However, it has been also reported that colonic diverticular hemorrhage generally stopped spontaneously, approximately 76%–91% of patients [10, 11]. Moreover, the rate of SRH identification with early colonoscopy has been still low, only 15%–42% [12–16], due to spontaneous hemostasis during colonoscopy and multiple diverticulum in several cases, which would result in ineffective intervention for colonic diverticular hemorrhage.

On these backgrounds, we performed follow-up assessments on the use of conservative treatment alone without performing early colonoscopy, except for cases with two signs of sustained bleeding: shock based on vital signs and extravasation on contrast-enhanced computed tomography (CECT). The aim of this study was to clarify the effectiveness of conservative treatment for colonic diverticular hemorrhage with respect to primary hemostasis and rebleeding.

## 2. Methods

### 2.1. Patients

This study included 142 consecutive patients who met the following criteria: (i) patients with bloody stools who visited as outpatients and were urgently hospitalized between April 2012 and December 2016; (ii) colonic diverticula were observed on colonoscopy or CECT; and (iii) other bleeding sources were ruled out via upper and lower gastrointestinal tract endoscopy or abdominal CECT. This study was approved by the institutional ethics review board at our hospital, and all patients gave informed consent about risks and benefits of their treatment depending on physical condition.

### 2.2. Flowchart of the Treatment Options

**Figure 1:**
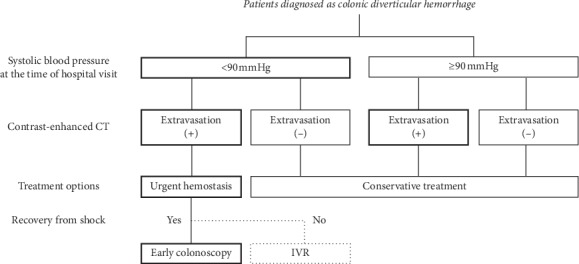
Flowchart of the treatment options for colonic diverticular hemorrhage. CT, computed tomography; IVR, interventional radiology. In case with both shock and extravasation on contrastenhanced computed tomography, early colonoscopy was performed. However, in case of uncontrolled hemorrhagic shock after initial treatment with fluid resuscitation, interventional radiology was considered for hemostasis. In other cases, conservative treatment with fasting and fluid administration was conducted.

The treatment options are shown in Figure 1. If shock (systolic blood pressure <90 mmHg) was observed at the time of hospital visit, we first performed fluid administration such as rapid infusion and blood transfusion to stabilize the general condition of the patient. All patients were then subjected to abdominal CECT to assess for extravasation within the bowel lumen, unless renal dysfunction was indicated based on the blood test results (estimated glomerular filtration rate >30 mL/min/1.73 m^2^). Whenever both shock and extravasation were observed at the time of hospital visit, early colonoscopy was definitely performed within 24 hours after admission. Additionally, if SRH was identified on early colonoscopy, hemostasis was firstly performed only by a clipping method. By contrast, if SRH was not identified due to spontaneous hemostasis during colonoscopy, follow-up observation was performed after the examination. In case of difficulty in recovering from shock or achieving endoscopic hemostasis, interventional radiology (IVR) was performed to stop bleeding by arterial embolization. Meanwhile, if vital signs were stable at the time of hospital visit or extravasation was not detected on CECT, conservative treatment was conducted without performing early colonoscopy. In these cases, elective colonoscopy was performed within 2 weeks after spontaneous hemostasis. In cases wherein CECT could not be performed due to renal dysfunction or allergy from the contrast agent, indications for early colonoscopy were determined based solely on the abovementioned guidelines on vital signs.

With regard to the indications of blood transfusion, patients with a serum hemoglobin (Hb) level <7 g/dL are considered for blood transfusion. However, in some patients with severe comorbidities or general conditions, such as shock, blood transfusion is considered even if the Hb level is less than 9 g/dL. Food intake was started if bloody stools had not been observed for >24 hours. The meals were gradually solidified each day and patients were discharged with a regular diet. Antithrombotic drugs were generally discontinued in patients with shock and immediately restarted after hemostasis, while antithrombotic drugs were not discontinued with stable vital signs. Signs of rebleeding were defined as the presence of fresh bloody stool along with low blood pressure (systolic blood pressure <90 mmHg) or a decrease in the Hb level of ≥2.0 g/dL. In case of rebleeding, colonoscopy was considered when both shock and extravasation on CECT were observed or continuous intermittent rebleeding was observed for ≥2 days. Early rebleeding was defined as rebleeding within 30 days after hospitalization, and recurrent bleeding as massive hematochezia more than 30 days after initial hospitalization, which resulted in second hospitalization. Presence or absence of recurrent bleeding was evaluated in all patients until February 2018 according to the following ways: (i) in case of outpatients in our hospital, it was evaluated at the day of onset or recent visit; (ii) in case of patients who were not followed in our hospital, it was evaluated by telephone call with questionnaire survey, which provided recent history about presence or absence of hospitalization due to hematochezia.

### 2.3. Routine Setting of Colonoscopy

Colonoscopy, including early colonoscopy, is performed after preparation with 2 L of polyethylene glycol (PEG). If patients have difficulty ingesting PEG due to poor general condition, either high-pressure enema or no preparation was carried out before colonoscopy. Carbon dioxide insufflation was used in all patients to reduce abdominal discomfort except for those with chronic obstructive pulmonary disease. PCF-Q260AZI (Olympus, Tokyo, Japan), which has a water jet system, was used for early colonoscopy with a cap attachment and PCF-Q260AZI or CF-H260AZI (Olympus, Tokyo, Japan) for elective colonoscopy.

### 2.4. Clipping

If SRH was identified, hemostasis was firstly performed only by a clipping method for all patients. Clips (HX-610-135; Olympus, Tokyo, Japan) were placed directly on the visible vessel or stigmata if possible. When direct placement was difficult because of a diverticular dome location, massive hemorrhage, or small diverticular orifice, indirect placement was performed with multiple clips in a zipper fashion [17, 18].

### 2.5. Interventional Radiology

IVR was performed in the femoral artery using 4-Fr Shepherd hook catheter, and nonionic contrast medium was injected in the superior mesenteric artery (5 mL/s) and inferior mesenteric artery (3 mL/s) to identify the bleeding sites. Once the bleeding sites were identified, a microcatheter was carefully advanced to the bleeding site, and arterial embolization was performed using coils.

### 2.6. Statistical Analysis

Mean ± standard deviation or percentage was used for all data. Categorical data were compared using the chi-square test or Fisher's exact test, and continuous data were compared using the Wilcoxon rank sum test. The relationship between necessity of early colonoscopy and backgrounds was examined using Fisher's exact test; multivariate logistic regression analyses were unsuitable and not used due to a small number of cases in the urgent hemostasis group. Kaplan–Meier method and the log-rank test were used in the time-to-event analysis in patients with recurrent bleeding, and the Cox proportional hazard model was performed to examine the factors affecting recurrent bleeding. In the statistical analysis, JMP (version 13; SAS Institute Inc., USA) was used, and a *P*-value <0.05 was considered statistically significant.

## 3. Results

### 3.1. Initial Hemostasis

Characteristics of the patients are shown in Table 1. Among the participants, 88 were males and 54 were females with an average age of 71.7 ± 12.7 (range: 34–94) years, and hemorrhagic shock was observed in 11.3% of the patients. The results are shown in Table 2. Of the 142 patients with colonic diverticular hemorrhage, 137 (96.5%) received conservative treatment, and only five (3.5%) underwent urgent hemostasis. In the conservative treatment group, 11 (8.0%) patients presented with shock at the time of hospital visit. However, spontaneous hemostasis was achieved in all patients by fasting and gut rest, and none of the patients required hemostasis by elective colonoscopy. Only one (0.7%) patient in the conservative treatment group died due to bacterial pneumonia, and not from hemorrhagic shock. Blood transfusion was performed in 60 (43.8%) patients, and the mean Hb level at the time of blood transfusion was 7.2 ± 1.3 g/dL, and the mean transfusion volume was 2.7 ± 4.0 units.

In contrast, in the urgent hemostasis group, early colonoscopy was performed in four of five patients and SRH was identified in three patients, resulting in successful hemostasis by clipping. Clips could be placed directly on SRH in two of the three patients and indirectly in one patient due to small diverticular orifice. In the other one of four patients, SRH could not be identified due to severe bleeding, and one patient could not receive early colonoscopy due to difficulty in recovering from shock. They received IVR, resulting in successful hemostasis. Consequently, hemostasis was achieved successfully in all patients and no patients died with hemorrhagic shock. In the urgent hemostasis group, bleeding sites were all located in the right colon and three of five patients took oral antithrombotic drugs.

The association between the necessity of early colonoscopy and the risk factors were examined (Table 3), and shock based on vital sings and extravasation on CECT at the time of hospital visit were only risk factors of early colonoscopy.

### 3.2. Incidence of Rebleeding after Treatment

#### 3.2.1. Early Rebleeding

Early rebleeding was not observed in all the patients in the urgent hemostasis group, whereas it was observed in 7.3% [95% confidence interval (CI): 4.0–12.9] of the patients in the conservative treatment group. There were no significant differences between the two groups. In addition, all the patients with early rebleeding achieved spontaneous hemostasis with conservative treatment. There was no significant relationship between early rebleeding and clinical backgrounds.

#### 3.2.2. Recurrent Bleeding

**Figure 2:**
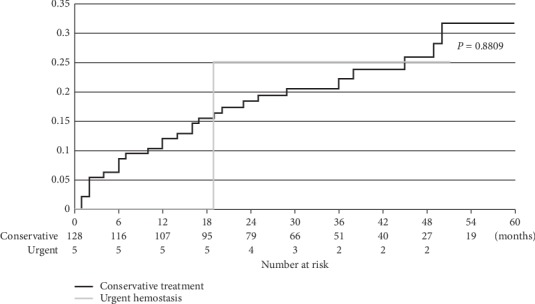
Recurrent bleeding rates after conservative treatment and urgent hemostasis. During a follow‐up period of 31.2 ± 18.4 months, the overall recurrent bleeding rate after conservative treatment and urgent hemostasis were 22.7% and 20.0%, respectively. The cumulative recurrent bleeding rate after conservative treatment was 12.1% after 1 year, 22.1% after 3 years, and 31.6% after 5 years.

In the conservative treatment group, recurrent bleeding was observed in 29 of 128 patients (22.7%; 95% CI: 16.3–30.6) during the observation periods of 31.3 ± 18.6 (14–68) months, excluding those with duplicated cases. It occurred 2–38 (12.4 ± 11.3) months after initial conservative treatment. The cumulative recurrent bleeding rates were 12.1%, 22.1%, and 31.6% in 1, 3, and 5 years, respectively (Figure 2). Meanwhile, recurrent bleeding in the urgent hemostasis group was observed in one of 5 patients (20.0%, 18 months after hemostasis) during observation periods of 29.5 ± 13.5 (18–51) months. In this case, as recurrent life-threatening hemorrhage occurred at the same site as previous treatment, elective surgical treatment was performed. There were no significant differences between the two groups in terms of recurrent bleeding rates.

Univariate analysis using the log-rank test showed that the recurrent bleeding rate was significantly higher in patients with a previous history of diverticular hemorrhage and oral administration of low-dose aspirin and thienopyridine derivative (Table 4). The cumulative recurrent bleeding rates in the group without a previous history of diverticular hemorrhage were 6.1%, 10.5%, and 12.0% in 1, 2, and 3 years, whereas those with a history of diverticular hemorrhage were 26.9%, 47.0%, and 58.7% in 1, 2, and 3 years, respectively (*P* < 0.0001). The cumulative recurrent bleeding rates in the non-antithrombotic drug group were 6.5%, 13.5%, and 17.9% in 1, 2, and 3 years, whereas those in the low-dose aspirin group were 18.8%, 28.4%, and 39.3% in 1, 2, and 3 years, respectively (*P*=0.0216), and those in the thienopyridine derivative group were 21.4%, 43.8%, and 53.1% in 1, 2, and 3 years, respectively (*P*=0.0021). Furthermore, in only patients who received dual antiplatelet therapy (DAPT), recurrent bleeding occurred in 5 of 9 patients (55.6%), and the cumulative recurrent bleeding rate was also significantly higher at 37.5% after 1 year and 81.3% after 2 years compared to the non-antithrombotic drug group (*P* < 0.0001).

Multivariate analyses using Cox proportional hazard model showed that patients with a previous history of diverticular hemorrhage (hazard ratio 5.63, 95% CI 2.68–12.0, *P* < 0.0001) or oral administration of thienopyridine derivative (hazard ratio 3.05, 95% CI 1.23–7.53, *P*=0.016) were significantly associated with recurrent bleeding.

### 3.3. Length of Hospital Stay and Cost

The length of fasting did not significantly differ between the conservative treatment and urgent hemostasis groups (4.1 ± 1.7 vs. 4.8 ± 1.9 days, *P*=0.3418). The hospitalization period of the conservative treatment group was significantly shorter than that of the urgent hemostasis group (11.3 ± 5.9 vs. 19.2 ± 21.4 days, *P*=0.0395). The hospitalization cost was significantly higher in the urgent hemostasis group compared to the conservative treatment group ($8237.28 ± 3211.87 vs. $4270.68 ± 2452.30, *P*=0.0001), even in the case except for the cost of IVR ($7169.06 ± 2168.09, *P*=0.0468).

## 4. Discussion

In Japan, colonic diverticular hemorrhage is increasingly common with aging population and westernization of dietary habits, and valuable treatment options should be fully discussed for the management of colonic diverticular hemorrhage. Currently, Japanese guidelines proposed early colonoscopy as the triage tool for patients with sustained bleeding although it remains controversial whether early colonoscopy would contribute to increase in identification of SRH or prevention of rebleeding [6]. On the contrary, our results in a real clinical setting suggested that colonic diverticular hemorrhage would stop spontaneously without any interventions, i.e., with only conservative treatment, except for the cases with shock based on vital signs and extravasation on CECT. In addition, interventions of urgent hemostasis contributed little to decrease in recurrent bleeding compared to conservative treatment.

Recently, a multicenter randomized control trial demonstrated that early colonoscopy for acute lower gastrointestinal bleeding did not improve the rate of SRH identification and reduce rebleeding [19], and this report would strongly support the results of our study. Moreover, the trial revealed that the rate of SRH identification was 21.3% (10/47). However, the primary endpoint of our study was a rate of successful hemostasis spontaneously by conservative treatment which was quite high of 96.5%. We hypothesized that most of SRH such as visible vessel and adherent clot in early colonoscopy might result in spontaneous hemostasis only with conservative treatment.

Most of diverticular hemorrhage, which is generally thought to be caused by a breakage in the vulnerable part of the vasa recta in protrusions, basically results in venous bleeding [20, 21] and would stop spontaneously without immediate hemostasis. In fact, previous reports have shown that spontaneous hemostasis occurred in 76%–91% of all cases [10, 11], and our result was also consistent with this principle. By contrast, some diverticular hemorrhage could be caused by arterial bleeding, which would require urgent hemostasis and have high risk of rebleeding even if it stops spontaneously. It should be crucial to assess whether hemorrhage was caused by arterial or venous bleeding.

In this study, the criteria for early colonoscopy were coexistence of shock at the time of hospital visit and extravasation on CECT, which were indicators of active bleeding. Although shock in patients with diverticular hemorrhage might contribute to temporary hypovolemic shock due to arterial bleeding, there is another possibility of vagal reflex in several cases. In addition, CECT, which could identify the localization of the bleeding sites [22, 23] and predict the risk of developing unstable vital signs after hospitalization, can detect bleeding as small as 0.5 mL/min [24, 25], which indicates that extravasation does not necessarily represent arterial bleeding. Therefore, we hypothesized that spontaneous hemostasis might be most likely achieved if only one of the two was observed, and conservative treatment could be the first choice of treatment in these cases. In fact, in these cases, spontaneous hemostasis was achieved with conservative treatment in all patients. By contrast, in case with both shock and extravasation on CECT, two of five patients suffered severe life-threatening hemorrhage which required hemostasis by IVR. Moreover, SRH could be all identified in the other three cases during early colonoscopy. Thus, we believe that the criteria of early colonoscopy used in this study would be highly likely to be appropriate, and the prospective study should be conducted with large sample size in the future.

Based on several meta-analyses, early colonoscopy for diverticular hemorrhage improved identification of SRH although it did not significantly improve mortality rate, rebleeding rate, and duration of hospital stay compared with those of elective colonoscopy cases [14, 26–28]. In addition, when performing early colonoscopy, preparation with PEG is basically indispensable in improving the rate of SRH identification [16, 29]. The majority of patients with diverticular hemorrhage are elderly and the ingestion of PEG forces a significant load in patients with a poor general condition. Considering the fact that spontaneous hemostasis occurred in most cases, the indication for early colonoscopy should be further narrowed down. If early colonoscopy was assumed to be performed in all cases, patients would then be transferred to facilities that could offer early colonoscopy, and the load in these facilities would rapidly increase.

It has been reported that the rate of early rebleeding within 30 days was 15%–30% in either the endoscopic hemostasis group or the conservative treatment group [14, 15, 30, 31], and the long-term recurrent bleeding rate was 14%–40% [10, 14, 18, 32, 33]. The early rebleeding rate in this study was favorable, i.e., approximately 7.3% in the conservative treatment group and 0% in the urgent hemostasis group. In the conservative treatment group, if rebleeding occurred, spontaneous hemostasis was again achieved by conservative treatment in all cases. When assessing risk factors for early rebleeding, neither conservative treatment nor urgent hemostasis was considered as a significant risk factor. Moreover, the severity of initial bleeding was not associated with early rebleeding. Meanwhile, in recurrent bleeding, the 5-year cumulative recurrent bleeding rate was 31.6% in the conservative treatment group, which was almost similar to the recurrent bleeding rates in institutions where hemostasis intervention was actively performed [18, 32, 34]. This result was consistent with previous reports showing that early colonoscopy did not contribute to the reduced incidence of recurrent bleeding [14]. With regard to the risk factors of recurrent bleeding, a previous history of diverticular hemorrhage as well as use of thienopyridine derivative was significantly associated with recurrent bleeding, although there was a report showing that the use of nonsteroidal anti-inflammatory drugs and antiplatelet drugs as well as high blood pressure increased the frequency of rebleeding [16, 33, 35, 36]. Moreover, in the present study, patients with DAPT had an extremely high recurrent bleeding rate, which was also anticipated to increase. Further studies on high-risk groups with recurrent bleeding must be conducted.

The median period of hospitalization in the conservative treatment group in the present study was 10 days, which was similar to the report from an institution that performed early colonoscopy for colonic diverticular hemorrhage in Japan [37]. Because the cost for hemostasis or the additional cost for hospitalization due to serious conditions is not involved in the conservative treatment, medical expenses for hospitalization are reduced. This was confirmed in our study even though the number of patients was relatively small. Issues related to medical expenses would vary depending on the circumstances of each country; however, because spontaneous hemostasis could be achieved without any other treatments in majority of the cases, there is no doubt that conservative treatment is superior to other treatments in terms of medical costs. On the other hand, periods of fasting in the conservative treatment group seemed long (4.1 days) and length of hospitalization was longer compared to that in the RCT report (11.3 days vs. 7.6 days) although subjects were different between the two studies [19]. These facts would be attributed to our study design of retrospective study; resumption of food intake and timing of discharge were decided at the individual discretion of bedside physicians. From the viewpoint of “budgeting,” a longer hospital stay can become a burden to hospital management. To solve the issue, a prospective study was conducted and is now ongoing (UMIN000028007).

This study has several strengths. First, conservative treatment without early colonoscopy would be a candidate for most patients, except for cases with both shock based on vital signs and extravasation on CECT. Second, conservative treatment without early colonoscopy would be fewer burdens on patients economically and physically compared to early colonoscopy. By contrast, our study has several limitations. First, this is a retrospective consecutive study, not a randomized controlled trial; the study was performed at a single facility, and the sample size was relatively small especially in the urgent hemostasis group. Our results should be certainly validated in a multicenter prospective study. Second, CECT was performed in all patients because it has the clinical benefits to identify bleeding sources including rectal ulcer, colorectal cancer, ischemic colitis, and so on, except for diverticular hemorrhage [22, 23]. The identification would allow for efficient clinical management depending on each bleeding source.

## 5. Conclusion

Conservative treatment without performing early colonoscopy was a useful therapeutic option for the patients with colonic diverticular hemorrhage to achieve spontaneous hemostasis, except for those with both shock and extravasation on CECT.

## Figures and Tables

**Table 1:** Characteristics of patients.

*Patients (n* *=* *142)*	
Age (years, mean ± SD)	71.7 ± 12.7 (34–94)
Sex (male/female)	88/54
Localization of diverticulum	
Right-sided	38 (26.8%)
Left-sided	14 (9.9%)
Bilateral	90 (63.4%)
Previous history of diverticular hemorrhage	38 (26.8%)
Patient on dialysis	11 (7.7%)
Patient with cirrhosis	3 (2.1%)
Medications	
Antithrombotic drugs (total)	56 (39.4%)
Aspirin	37 (26.1%)
Thienopyridine derivative	17 (12.0%)
Anticoagulants	23 (16.2%)
Dual antiplatelet therapy	10 (7.0%)
NSAIDs	14 (9.6%)
Systolic blood pressure (mmHg, mean ± SD)	127.2 ± 30.7
Hemorrhagic shock	16 (11.3%)
Heart rate (bpm, mean ± SD)	86.6 ± 20.5
Loss of consciousness	17 (12.0%)
Extravasation on CECT^*∗*^	18 (18.4%)
Laboratory data	
Hemoglobin level (g/dL, mean ± SD)	10.5 ± 2.5
White blood cell count (×10^3^/mm^3^, mean ± SD)	8.1 ± 2.9
Platelet count (×10^4^/mm^3^, mean ± SD)	20.4 ± 6.1
UN/Cre ratio (mean ± SD)	23.0 ± 10.4
Albumin level (g/dL, mean ± SD)	3.5 ± 0.5
PT-INR (mean ± SD)	1.2 ± 0.7

SD, standard deviation; NSAIDs, nonsteroidal anti-inflammatory drugs; CECT, contrast-enhanced computed tomography; UN/Cre, urea nitrogen/creatinine; PT-INR, international normalized ratio of prothrombin time. ^*∗*^CECT was performed in ninety-eight patients.

**Table 2:** Treatment outcomes.

	Conservative treatment (*n* = 137)	Urgent hemostasis (*n* = 5)	*P*-value
Hemostasis	137 (100%)	5 (100%)^*∗*^	
Mortality			
Bleeding	0 (0%)	0 (0%)	0.978
Others	1 (0.7%)	0 (0%)	
Blood transfusion	60 (43.8%)	5 (100%)	0.003
Units of blood (mean ± SD)	2.7 ± 4.0	10.0 ± 3.3	0.0002
Hb level during transfusion (g/dL, mean ± SD)	7.2 ± 1.3	7.0 ± 1.5	
Hemorrhagic shock (systolic BP < 90 mmHg)	11 (8.0%)	5 (100%)	<0.0001
Periods of fasting (days, mean ± SD)	4.1 ± 1.7 (1–9)	4.8 ± 1.9 (4–7)	0.342
Length of hospitalization (days, mean ± SD)	11.3 ± 5.9 (3–34)	19.2 ± 21.4 (9–30)	0.040
Hospitalization costs (USD, mean ± SD)	4270.68 ± 2452.30	8237.28 ± 3211.87 7169.06 ± 2168.09^*∗∗*^	0.0001
			0.047
Early rebleeding	10 (7.3%)^*∗∗∗*^	0 (0%)	0.690
Recurrent bleeding	29 (22.7%)	1 (20%)	0.685

SD, standard deviation; Hb, hemoglobin; BP, blood pressure; *USD,* the United States dollar. ^*∗*^Three of five patients underwent endoscopic hemostasis, and the other two patients underwent transcatheter arterial embolization, resulting in successful hemostasis in all patients. ^*∗∗*^Except the two patients who received interventional radiology treatment, ^*∗∗∗*^all patients achieved spontaneous hemostasis after early rebleeding.

**Table 3:** Factors associated with urgent hemostasis.

	Conservative treatment (*n* = 137)	Urgent hemostasis (*n* = 5)	*P*-value
Age (years, mean ± SD)	71.6 ± 12.8	74.2 ± 11.5	0.652
Sex (male)	84 (61.3%)	4 (80.0%)	0.650
Previous history of diverticular hemorrhage	36 (26.3%)	2 (40.0%)	0.610
Patient on dialysis	11 (8.3%)	0 (0%)	0.999
Patient with cirrhosis	3 (2.2%)	0 (0%)	0.999
Medications			
Antithrombotic (total)	53 (38.9%)	3 (60.0%)	0.383
Aspirin	35 (25.5%)	2 (40.0%)	0.605
Thienopyridine derivative	16 (11.7%)	1 (20.0%)	0.477
Dual antiplatelet therapy	9 (6.6%)	1 (20.0%)	0.310
Anticoagulants	21 (15.3%)	2 (40.0%)	0.189
NSAIDs	13 (9.5%)	1 (20.0%)	0.410
Systolic blood pressure <90 (mmHg)	11 (8.3%)	5 (100%)	<0.0001
Heart rate (bpm, mean ± SD)	87.0 ± 20.4	74.2 ± 22.8	0.184
Loss of consciousness	17 (12.4%)	0 (0%)	0.999
Extravasation on CECT	13 (14.0%)^*∗*^	5 (100%)	0.0001
Localization of diverticulum (right-sided/left-sided/bilateral)	34/14/89	4/0/1	0.004
Laboratory data			
Hemoglobin level (g/dL, mean ± SD)	10.5 ± 2.5	10.9 ± 3.4	0.696
WBC count (×10^3^/mm^3^, mean ± SD)	8.1 ± 2.9	8.1 ± 4.0	0.988
Platelet count (×10^4^/mm^3^, mean ± SD)	20.5 ± 6.2	18.9 ± 2.0	0.584
UN/Cre ratio (mean ± SD)	23.1 ± 10.5	19.1 ± 3.4	0.390
Albumin level (g/dL, mean ± SD)	3.5 ± 0.5	3.2 ± 0.5	0.211
PT-INR (mean ± SD)	1.2 ± 0.7	1.5 ± 0.6	0.429

SD, standard deviation; NSAIDs, nonsteroidal anti-inflammatory drugs; CECT, contrast-enhanced computed tomography; WBC, white blood cells; UN/Cre, urea nitrogen/creatinine; PT-INR, international normalized ratio of prothrombin time. ^*∗*^CECT was performed in ninety-three patients in the conservative treatment group.

**Table 4:** Factors associated with recurrent bleeding after spontaneous hemostasis.

	Recurrent bleeding	Nonbleeding	Univariate analysis	Multivariate analysis	
	(*n* = 29)	(*n* = 99)	*P*-value	HR (95%CI)	*P*-value
Age (years, mean ± SD)	73.8 ± 11.8	70.5 ± 13.4	0.114		
Sex (male)	20 (69.0%)	59 (59.6%)	0.414		
Previous history of diverticular hemorrhage	15 (51.7%)	12 (12.1%)	<0.0001	5.63 (2.68–12.0)	<0.0001
Patient on dialysis	3 (10.3%)	7 (7.1%)	0.234		
Patient with cirrhosis	1 (3.4%)	1 (1.0%)	0.315		
Medications					
Aspirin	12 (41.4%)	21 (21.2%)	0.022	1.80 (0.84–3.88)	0.133
Thienopyridine derivative	7 (24.1%)	8 (8.1%)	0.002	3.05 (1.23–7.53)	0.016
Anticoagulants	7 (24.1%)	14 (14.1%)	0.216		
NSAIDs	3 (10.3%)	10 (10.1%)	0.607		
Systolic blood pressure <90 (mmHg)	2 (6.9%)	9 (9.1%)	0.696		
Heart rate (bpm, mean ± SD)	84.4 ± 19.8	88.6 ± 20.9	0.865		
Loss of consciousness	4 (13.8%)	13 (13.1%)	0.699		
Extravasation on CECT	2 (11.8%)^*∗*^	10 (14.1%)^*∗*^	0.741		
Localization of diverticulum (right-sided/left-sided/bilateral)	7/2/20	27/12/60	0.119		
Laboratory data					
Hemoglobin level (g/dL, mean ± SD)	9.7 ± 2.5	10.8 ± 2.4	0.059		
WBC count (×10^3^/mm^3^, mean ± SD)	7.1 ± 2.5	8.5 ± 2.9	0.177		
Platelet count (×10^4^/mm^3^, mean ± SD)	20.3 ± 6.4	20.7 ± 6.3	0.970		
UN/Cre ratio (mean ± SD)	23.1 ± 9.4	23.1 ± 10.9	0.948		
Albumin level (g/dL, mean ± SD)	3.5 ± 0.6	3.5 ± 0.5	0.793		
PT-INR (mean ± SD)	1.2 ± 0.6	1.2 ± 0.8	0.326		

Follow-up period: 31.3 ± 18.6 months. HR, hazard ratio; CI, confidence interval; SD, standard deviation; NSAIDs, nonsteroidal anti-inflammatory drugs; CECT, contrast-enhanced computed tomography; WBC, white blood cells; UN/Cre, urea nitrogen/creatinine; PT-INR, international normalized ratio of prothrombin time. ^*∗*^CECT was performed in seventeen patients in the recurrent bleeding group and in seventy-one patients in the nonbleeding group.

## Data Availability

The data used to support the findings of this study are included within the article.
